# Responding to Reviewers

**DOI:** 10.1093/geroni/igaa057.3163

**Published:** 2020-12-16

**Authors:** Deborah Carr

**Affiliations:** Boston University, Boston, Massachusetts, United States

## Abstract

An invitation to “revise and resubmit” one’s manuscript, especially for a highly competitive journal, is an important achievement. However, it is not a guarantee that a revised manuscript will ultimately be accepted for publication. I will provide insights into what differentiates a “major” versus “minor” R&R invitation, will provide background on how editors and reviewers arrive at these decisions, and will provide detailed tips for crafting a revision memo and revised manuscript that has an excellent chance of being accepted for publication (while staying within a journal’s word count limits). I will also offer suggestions for navigating revisions when reviewers offer discrepant feedback, or make suggestions that may not ultimately enhance the quality or impact of the manuscript.

